# Specialties

**Published:** 1864-05

**Authors:** 


SPECIALTIES.—The question of specialties has nearly found its level in this country, and has been settled by admitting them in the bosom of the hospitals and centres of instruction, where they can serve purposes of progress and education within salutary limits and subject to the regulations of the general body. Left to themselves they grow rank and overrun the place in lawless outgrowths. In America, the professors of specialties have adopted the fashion of advertising. Thus we read that "Dr. Elsberg, Lecturer on the Laryngoscope and Diseases of the Larynx and Throat in the University of New York, devotes himself specially to the treatment of the larynx and neighboring organs—office hours from four to six P. M.;" which announcement, with others similar to it, appears in large capitals, variously spaced, in the advertising columns of the principal weekly periodicals of America. Here there could not be any difference of opinion about the exceedingly gross impropriety of such a proceeding. However, various standards rule in different countries, and possibly the American profession may find as much reason to wonder at irregularities that we tolerate, as we do at the lax proceedings which their professional code admits.—*London Lancet.*

